# Introduction of a National Minimum Wage Reduced Depressive Symptoms in Low‐Wage Workers: A Quasi‐Natural Experiment in the UK

**DOI:** 10.1002/hec.3336

**Published:** 2016-04-04

**Authors:** Aaron Reeves, Martin McKee, Johan Mackenbach, Margaret Whitehead, David Stuckler

**Affiliations:** ^1^ Department of SociologyUniversity of OxfordOxfordUK; ^2^ Department of Public Health and PolicyLSHTMLondonUK; ^3^ Department of Public HealthErasmus MCRotterdamNetherlands; ^4^ Department of Public Health and PolicyUniversity of LiverpoolLiverpoolUK; ^5^ Present address: International Inequalities InstituteLondon School of Economics and Political ScienceLondonUK

**Keywords:** minimum wage, natural experiments, health policy, GHQ caseness

## Abstract

Does increasing incomes improve health? In 1999, the UK government implemented minimum wage legislation, increasing hourly wages to at least £3.60. This policy experiment created intervention and control groups that can be used to assess the effects of increasing wages on health. Longitudinal data were taken from the British Household Panel Survey. We compared the health effects of higher wages on recipients of the minimum wage with otherwise similar persons who were likely unaffected because (1) their wages were between 100 and 110% of the eligibility threshold or (2) their firms did not increase wages to meet the threshold. We assessed the probability of mental ill health using the 12‐item General Health Questionnaire. We also assessed changes in smoking, blood pressure, as well as hearing ability (control condition). The intervention group, whose wages rose above the minimum wage, experienced lower probability of mental ill health compared with both control group 1 and control group 2. This improvement represents 0.37 of a standard deviation, comparable with the effect of antidepressants (0.39 of a standard deviation) on depressive symptoms. The intervention group experienced no change in blood pressure, hearing ability, or smoking. Increasing wages significantly improves mental health by reducing financial strain in low‐wage workers. © 2016 The Authors. *Health Economics* published by John Wiley & Sons Ltd.

## 1 Introduction

Does increasing income improve people's health? The statistical association between poverty and worse health outcomes is well‐known, including self‐reported health (Benzeval and Judge, 2001), access to healthcare (Lorant *et al*., 2003), and mental ill health (Costello *et al*., 2003; Krieger *et al*., 1997). Moreover, whether or to what extent these associations reflect a causal effect of income on health is unclear. A recent systematic review of the impact of income changes on self‐reported health found ‘mixed results and many… studies of poor quality’ (Gunasekara *et al*., 2011), with marked heterogeneity across study types (Apouey and Clark, 2015), contexts (Sacker *et al*., 2007), and sample populations (Frijters and Ulker, 2008). The health effects of increases in income depend on how it is used, potentially not only to increase access to healthcare, leisure, or physical activity, which may be health promoting (Marmot, 2002; McCarrier *et al*., 2011), but also to purchase alcohol (Bor *et al*., 2013), tobacco (Chaloupka *et al*., 2011), and unhealthy dietary products (Jensen and Miller, 2008; Jetter and Cassady, 2006). Psychological well‐being may be particularly susceptible to income changes, because poverty generates financial strain, a known correlate of major depression (Taylor *et al*., 2011). Further, the effect of income on health is likely to be non‐linear, so that the effects on low‐income groups may be greater than those better off (Jones and Wildman, 2008; Leigh and Du, 2012; Mackenbach *et al*., 2005). Summarising the state of the evidence, a recent review concludes that “a definite causal relationship between income and health has…not yet been established” (Gunasekara *et al*., 2011).

Observational studies are limited by potential confounding factors. Often, these studies lack a well‐specified counterfactual for what would have occurred in the absence of an increase in income (if *A* had not occurred, then *B* would not have occurred) (Heckman, 2008; Morgan and Winship, 2007; Pearl, 1995; Pearl, 2010; Pearl, 2000). Reverse causality also constrains causal inference, as persons who develop ill health may experience reduced incomes, rather than vice versa (Deaton, 2003; Smith, 1999). Randomised controlled trials are used to help answer questions of causality but are rare in social and economic policy, with pragmatic and ethical barriers often cited as reasons for not conducting such trials. Importantly, however, the artificial setting of a trial may limit external validity as the contextual factors may modify interventions in unanticipated ways (Petticrew *et al*., 2013). To overcome some of these limitations, recent studies have focussed on exploiting ‘natural experiments’ (Angrist and Pischke, 2009; Craig *et al*., 2012; Dunning, 2008; Dunning, 2012; Petticrew *et al*., 2005; Robinson *et al*., 2009; Sekhon and Titiunik, 2012; Reeves *et al*., 2016) or specific exogenous changes *in milieu* that assign individuals to either the intervention or the control groups through a process that is random or is ‘as‐if random’, such as winning a lottery or naturally occurring variation in the roll‐out of policy interventions (Dunning, 2012; Morgan and Winship, 2007).

In 1999, the UK government introduced National Minimum Wage legislation, mandating a wage floor of £3.60 per hour. Employers who did not pay minimum wages faced a £5000 fine (Ipsos, 2012). For workers earning below the threshold, the wage increase corresponded to an average 30% pay rise (Low Pay Commission, 2000). However, those people earning above the threshold were likely unaffected, and, as compliance was imperfect, not all low‐wage groups actually received higher wages. Thus, the UK National Minimum Wage intervention creates a rare natural experiment to be exploited (Leigh, 2007; Neumark and Wascher, 2001; Stewart and Swaffield, 2002; Stewart, 2004b).

Although many studies have examined the economic effects of minimum wages, there is ongoing debate about its net effects and potential unintended consequences. This debate concentrates on, in particular, whether wages also rise for those above the minimum wage threshold, whether the numbers of hours worked decrease, and whether minimum wages increase risks of job loss. Taking one example, US studies of minimum wages suggest that they increased wages for those just above the minimum wage threshold, whereas in the UK, there is no evidence of such spillovers, although the contextual factors that underlie such international differences are not well understood (Dickens and Manning 2004a; Dickens and Manning 2004b; Neumark *et al*., 2004; Stewart, 2012).

To our knowledge, however, no study has investigated the *health effects* of the UK National Minimum Wage. Several US studies have found that minimum wages corresponded to lower unmet medical need and obesity rates (Kim and Leigh, 2010; McCarrier *et al*., 2011), and others have investigated effects of earned income tax credits on smoking and low birth weight among recipients' children, using natural experiment methods (Averett and Wang, 2013; Hoynes *et al*., 2015). Building on these prior studies, we test whether increases in wages among low‐wage groups had a positive health effect, evaluating the natural policy experiment created by the National Minimum Wage with longitudinal data from the British Household Panel Survey.

One plausible and potentially significant mechanism by which increasing wages may yield health (as well as potentially economic) benefits is by reducing financial strain. This occurs when people's subjective assessment of their financial situation improves—which is one risk factor for poor mental health. Longitudinal evidence from the UK and Australia find that when people transition into unaffordable housing, they become more likely to experience poorer mental health (Bentley *et al*., 2011; Bentley *et al*., 2015; Taylor *et al*., 2007). Similarly, those who experienced financial difficulties over an 18‐month period became more likely to suffer from common mental health problems than those who did not experience financial difficulties (Skapinakis *et al*., 2006). Given this evidence, we would expect that those who receive the minimum wage will experience a greater reduction in financial strain than those who do not and that this change in financial strain will mediate the association between the minimum wage and health.

Specifically, in this study, we test the hypothesis that increasing wages leads to significant improvements in mental health, or psychiatric ‘caseness’, by reducing financial strain by comparing the effects of the wage increase on those who receive it (intervention group) with those persons who did not receive a wage boost (control group).

## 2 Methods

### 2.1 Data

The British Household Panel Survey (BHPS) is a nationally representative longitudinal survey of 5500 households and ~10 000 individuals, covering the years from 1991 to 2009; details of the survey have been described elsewhere (Lynn *et al*., 2006). Briefly, households were randomly selected from 250 primary sampling units (postcode sectors) and subsequently stratified by socio‐economic factors, oversampling impoverished groups (Lynn *et al*., 2006). The same individuals were interviewed annually in successive ‘waves’ of the survey. Data were drawn for the period just prior to the introduction in 1998 (pre‐intervention, wave 8) and after in 1999 (post‐intervention, wave 9), although we also consider the longer term trends for these groups between 1994 and 2001. Men and women who were under 22 were excluded, because the full rate National Minimum Wage applied only to persons over that age. For those aged 18 to 21, there was a ‘special development rate’ set at £3.00 per hour; however, we removed this sub‐group (*n* = 28) because the impact is likely to differ from that in older persons (Low Pay Commission, 2000). Individuals aged 16–17 eventually became eligible for the minimum wage but not until 2004 and so are also excluded from the analysis here. Persons over age 59 were excluded because many would have become eligible for pensions. Additionally, persons who were not working at least 1 h per week in both 1998 and 1999 were excluded to separate the effects of wage increases from those of finding employment. The National Minimum Wage came into effect in April 1999; data collection for the BHPS in the wave prior to this began in September of 1998, and by the end of January 1999, 99% of the sample had been interviewed. This wave constitutes the before‐intervention observation. Thus, wave 9, which began in September 1999, constitutes the first post‐intervention observation (Morgan and Winship, 2007). While there was attrition in the full sample, there was zero attrition in either the treatment or control groups between 1998 and 1999 among those who participated in the survey in 1998 (i.e. the analytic sample) (Noah Uhrig, 2008).

### 2.2 Health outcomes

Based on previous literature, we are primarily interested in examining the impact of the introduction of the minimum wage on mental health outcomes. The probability of having a mental health problem was assessed using the General Health Questionnaire (GHQ), which we have treated as a continuous variable. The GHQ is a reliable mental health scale, robust to retest effects, and a good predictor of psychological problems, including depression (Argyle, 1987; Goldberg *et al*., 1997; Goldberg, 1978; McCabe *et al*., 1996; Pevalin, 2000). Although it comes in variants with different numbers of items, the BHPS used the 12‐item version. To facilitate interpretation, the indicator was positively coded so that high scores correspond to reduced likelihood of a mental health problem (improved mental health) (refer to Box 1). Although the GHQ is now used mainly to dichotomise individuals into cases or non‐cases of mental distress or disorder, an early evaluation of the properties of the 30, 28, and 12‐item variants found ‘support for the treatment of GHQ scores as a continuous variable’ (Banks, 1983). Moreover, the cut‐point used in contemporary studies varies considerably, as can be seen from a selection of recent publications, each using a different cut‐point (Adebowale and Adelufosi, 2013; Bianchini *et al*., 2013; Clarke *et al*., 2013; Reang and Bhattacharjya, 2013), and other researchers do use the score as a continuous variable (Theofilou, 2011) (in some cases in addition to a dichotomised one) (Bertotti *et al*., 2013) or have created a continuous scale from a sub‐set of questions (Risberg and Jacobsen, 2003). We also examined two specific components of the GHQ‐12 pertinent to the policy intervention, including whether the respondents have felt ‘constantly under strain’ or ‘unhappy or depressed’, as well as a BHPS measure of self‐reported depression.

In addition to mental health, we also examine the association between the introduction of the minimum wage and changes in physical health and health behaviours. To assess physical health, we included BHPS questions on whether respondents report chronic conditions, such as hearing difficulties. These measures are used as so‐called falsification tests because the introduction of the minimum wage would not plausibly change these health outcomes, at least not in the short term. We also examine other health measures that might be sensitive to short‐term fluctuations in income: (1) self‐reported diagnosis of elevated blood pressure, which may be responsive to short‐term fluctuations in stress, and (2) the number of cigarettes smoked per day among current smokers, which may increase if the minimum wage increases disposable income or may decrease if smoking is considered an ‘inferior good’ (Blanchflower and Oswald, 2008; Chaloupka and Warner, 2000).

**Box 1: Description of health outcomes and financial difficulties**

*GHQ*: GHQ caseness scale is a 13‐point version of the GHQ scale capturing general mental health from a battery of 12 questions (1—worst to 13—best).

*Constantly under strain*: Have you recently felt constantly under strain? Not at all or no more than usual = 0, rather more or much more = 1.

*Anxiety/depression*: Do you have any of the health problems or disabilities listed on this card? No = 0, yes = 1.

*Unhappy or depressed*: Have you recently been feeling unhappy or depressed? Not at all or no more than usual = 0, rather more or much more = 1.

*Hearing problems*: Do you have any of the health problems or disabilities listed on this card? No = 0, yes = 1.

*Blood pressure*: Do you have any of the health problems or disabilities listed on this card? Heart/blood pressure or blood circulation problems. No = 0, yes = 1.

*Number of cigarettes*: Approximately how many cigarettes a day do you usually smoke, including those you roll yourself?

*Current financial hardship*: How well would you say you yourself are managing financially these days? Would you say you are....? 1 = Living comfortably, 2 = doing alright, 3 = just about getting by, 4 = finding it difficult, 5 = finding it very difficult.

### 2.3 Random intervention assignment pattern

The intervention group comprises those who earned less than £3.60 per hour in 1998 and who then earned between £3.60 and £4.00 per hour in 1999. We compared health changes in people who received a wage increase (intervention group, *n* = 63) to those who did not because either (1) they were ineligible as their hourly wage was between 100 and 110% of the minimum wage, at £3.60 to £4.00 per hour (control group 1, *n* = 107), and (2) they were eligible but their firms did not comply, that is, their wages remained below the national minimum (control group 2, *n* = 109). We select the intervention group based on post‐intervention wages because we assume that any increase in earnings above 110% of the minimum wage is likely to be because of reasons other than the intervention (such as a job promotion or transition). This approach differs from previous intention‐to‐treat analyses that examine the impact on the policy change on those who were earning less than £3.60 per hour at baseline. This restriction, especially for control group 2, may create some selection bias, because psychologically fragile people may be more likely to be exploited by their firms and so not receive the National Minimum Wage. We expect this bias to be relatively small because the decision to introduce the minimum wage is made at the firm level and not at the individual level; nonetheless, as a robustness check, we test this possibility.

Wages were measured as self‐reported gross monthly income from the respondent's main job, divided by the number of hours worked in their main job, including overtime, to calculate hourly wages. In about one third of cases, self‐reported income was matched to payslips, providing further validation of earnings (Jenkins, 2010). Assignment to the intervention or control group can be considered as‐if random if members of both groups are comparable on other covariates (such as age). We use a series of statistical tests to examine this as‐if random assignment procedure (refer to Box 2 and Table 1). These tests cannot prove that the assignment pattern is as‐if random, but the results are consistent with that assumption.

**Box 2: Pre‐intervention comparisons**

If it is to identify causal effects, the natural policy experiment should simulate a randomised trial, so that all differences between the intervention and control groups apart from the wage increase are random. We argue this minimum wage natural policy experiment approximates a randomised trial, and therefore, we test whether the as‐if random aspect of this natural experiment is violated. Often, this occurs when subjects can self‐select into the intervention group. While in theory it is possible that persons above the income threshold could choose to move into a lower income group, this is very unlikely. Another possibility is that persons in the non‐compliance control group had differing working environments that may have resulted in prior health differences, but we found no significant difference in a range of such measures (Table 1). While it has been argued that the National Minimum Wage may have increased the latter group's risk of unemployment or led to wage reductions, neither has been observed (Stewart 2004b). We further tested whether the intervention and two control groups differed with respect to age, education, and other socio‐demographic factors prior to the introduction of the National Minimum Wage. None of the pre‐intervention covariates significantly differ at *α* = 0.05 in the non‐compliance sample. In the preceding and succeeding sample, we find that those earning more than £3.60 per hour in 1998 were more likely to be in the service class, less likely to be satisfied with their job, and had higher incomes than those earning less than £3.60 per hour.

**Table 1:** As‐if randomisation tests for intervention and control groups, prior to the introduction of the National Minimum Wage, 1998

	Intervention group (*n* = 63)	Control group 1 (*n* = 107)			Control group 2 (*n* = 109)		
	Mean (std. dev.)	Mean (std. dev.)	Intervention control (std. error)	*p* value	Mean (std. dev.)	Intervention control (std. error)	*p* value
*Socio‐demographic*							
Sex (female = 1)	0.87 (0.34)	0.76 (0.42)	0.097 (0.059)	0.099	0.85 (0.36)	0.020 (0.054)	0.72
Age	39.24 (11.32)	38.23 (11.90)	1.00 (1.83)	0.58	39.61 (10.80)	−0.38 (1.76)	0.83
Labour income (£ per month)	398.00 (222.66)	613.98 (285.27)	−215.98** (39.34)	<0.001	330.28 (205.99)	67.72 (34.30)	0.051
Hourly wage (£ per hour)	3.00 (0.49)	3.80 (0.11)	−0.79** (0.06)	<0.001	2.85 (0.65)	0.16 (0.046)	0.08
Post‐secondary education = 1^a^	0.36 (0.48)	0.49 (0.50)	−0.14 (0.093)	0.15	0.38 (0.49)	−0.032 (0.091)	0.73
Married = 1	0.57 (0.49)	0.60 (0.49)	−0.027 (0.079)	0.74	0.60 (0.49)	−0.024 (0.079)	0.75
							
*Pre‐intervention health status*							
General Health Questionnaire	11.05 (3.06)	11.36 (2.53)	−0.32 (0.46)	0.49	11.33 (2.98)	−0.28 (0.48)	0.56
Smoking^a^	17.00 (6.12)	14.95 (6.79)	2.05 (1.50)	0.18	17.76 (9.33)	−0.76 (1.67)	0.65
Financial strain	0.095 (0.30)	0.037 (0.19)	0.058 (0.042)	0.18	0.046 (0.21)	0.049 (0.042)	0.25
Blood pressure	0.11 (0.32)	0.056 (0.23)	0.055 (0.046)	0.23	0.11 (0.31)	0.0010 (0.050)	0.98
Hearing problems	0.048 (0.21)	0.037 (0.19)	0.010 (0.033)	0.76	0.037 (0.19)	0.011 (0.032)	0.74
Depression	0.25 (0.44)	0.25 (0.44)	−0.0016 (0.070)	0.98	0.23 (0.42)	0.025 (0.069)	0.72
Anxiety/depression	0.079 (0.27)	0.056 (0.23)	0.023 (0.041)	0.57	0.083 (0.28)	−0.0032 (0.043)	0.94
							
*Housing status*							
Owned home outright = 1	0.14 (0.35)	0.12 (0.33)	0.021 (0.054)	0.70	0.17 (0.38)	−0.031 (0.058)	0.59
Mortgage = 1	0.44 (0.50)	0.50 (0.50)	−0.060 (0.079)	0.45	0.36 (0.48)	0.087 (0.078)	0.27
Social renters = 1	0.35 (0.48)	0.26 (0.44)	0.088 (0.074)	0.24	0.29 (0.46)	0.056 (0.075)	0.46
Private renters = 1	0.048 (0.21)	0.093 (0.29)	−0.046 (0.039)	0.24	0.11 (0.31)	0.062 (0.040)	0.12
Joint *F*‐test for housing status				0.72			0.65
							
*Employment*							
Job hours	26.41 (13.55)	29.67 (11.95)	−3.46 (2.06)	0.096	24.65 (14.11)	1.76 (2.18)	0.42
Financial situation: ‘getting by’ = 1	0.29 (0.46)	0.31 (0.46)	−0.022 (0.073)	0.76	0.24 (0.43)	0.047 (0.071)	0.50
Job satisfaction	5.83 (1.16)	5.38 (1.29)	0.44* (0.19)	0.023	5.63 (1.29)	0.19 (0.19)	0.32
Full‐time employment = 1	0.51 (0.50)	0.33 (0.48)	0.17 (0.095)	0.07	1.61 (0.49)	0.10 (0.08)	0.19
Social class: service = 1	0.016 (0.13)	0.11 (0.32)	−0.096** (0.035)	0.0060	0.064 (0.25)	−0.048 (0.028)	0.091
Social class: routine non‐manual = 1	0.37 (0.49)	0.33 (0.47)	0.038 (0.076)	0.62	0.36 (0.48)	0.0073 (0.077)	0.92
Social class: routine manual = 1	0.38 (0.49)	0.47 (0.50)	−0.086 (0.078)	0.27	0.53 (0.50)	−0.15 (0.079)	0.055
Joint *F*‐test for social class				0.42			0.19

*p* value is calculated using two‐tailed *t*‐test assuming unequal variances. Hourly incomes are all below £4.00 per hour. Higher GHQ score captures better health (1 = worst, 13 = best).

a Restricted sample size in control group 1. Smoking: intervention *n* = 35, control *n* = 42. Post‐secondary education: intervention *n* = 42, control *n* = 88. Full‐time employment: intervention *n* = 63, control *n* = 45. Restricted sample size in control group 2. Smoking: intervention *n* = 35, control *n* = 51. Post‐secondary education: intervention *n* = 42, control *n* = 90. Full‐time employment: intervention *n* = 63, control *n* = 108.

* *p* < 0.05.

** *p* < 0.01.

### 2.4 Statistical model

**Box 3: Difference‐in‐difference modelling framework**

This modelling framework estimates differences in the health outcomes between 1998 and 1999 for both the intervention group and the control group. Thus, to estimate the health change in the intervention group, we sequentially estimate the ‘intervention effect on those receiving a wage increase’,

(3.1) $$\Delta H_{\text{wageincrease}}=\alpha +\beta_{\text{wageincrease}}\text{National Minimum Wage}\;+\varepsilon \text{,}$$

and the ‘intervention effect on the control group’,

(3.2) $$\Delta H_{\text{control}}=\alpha +\beta_{\text{control}}Non-\text{recipients of National Minimum Wage}\;+\varepsilon$$

Changes in this latter group reflect common background trends, such as those arising from contemporary changes in the labour market and macroeconomic circumstances. Thus, the intervention effect is the difference between these two observed effects, yielding the main difference‐in‐difference estimator (Angrist and Pischke 2009)

(3.3) $$\text{Intervention effect}\;=\beta_{\text{wageincrease}}–\beta_{\text{control}}$$

To evaluate the average intervention effect, we assessed the difference‐in‐difference between the observed within‐individual changes in health outcomes (*H*) over time in the intervention group compared with those in the control groups (refer to Box 3 for more details):

(1) $$\text{Intervention effect}\;=\;\Delta H_{\text{wageincrease}}-\;\Delta H_{\text{control}}\text{.}$$

To estimate the intervention effect, we used both differenced models and fixed‐effects regression models including an interaction term between a period dummy and an intervention indicator. Both sets of models produced very similar results, in part because they both adjust for individual‐specific differences that are constant over time. Using regression models, we further adjusted models for age, sex, social class (measured with the National Statistics Socio‐Economic Classification, a commonly used measure of occupation stratification), and education, which were not plausibly affected by the intervention (King *et al*., 2000). To test whether the pathway between the minimum wage and mental health problems (measured by GHQ‐12 as ‘psychiatric caseness’) is influenced by financial strain, we further examine whether receiving the wage increase because of the minimum wage legislation affects current financial strain (Taylor *et al*., 2011) (‘How well… are [you] managing financially these days?’) and whether the effect of the minimum wage on health is attenuated when this measure of financial strain is included in the difference‐in‐difference model. Additionally, we assess whether the observed effects were sustained in time periods after the initial year of intervention. We also compare the estimated effect sizes observed using the natural experimental approach with identification based on traditional multivariable regression models. All models were analysed in STATA v12.1.

## 3 Results

### 3.1 Health effects of the National Minimum Wage

**Figure 1:**
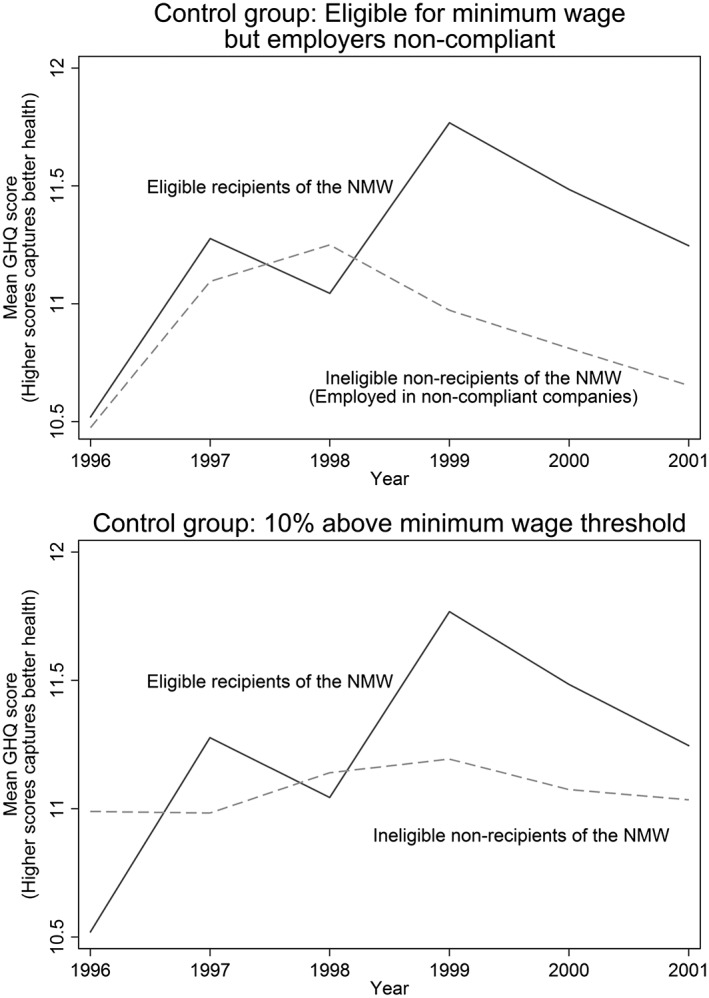
Observed differences in mean General Health Questionnaire scores between intervention and control groups, 1996–2001

Figure 1 depicts the observed differences in means in the intervention group and both control groups. In 1998, prior to the introduction of the National Minimum Wage, the recipients and control group had similar psychiatric caseness scores in the GHQ, physical health, and consumption of alcohol and tobacco (Table 1). As shown in the figure, after the introduction of the National Minimum Wage, recipients of the wage increase report significantly improved psychiatric caseness while eligible non‐recipients did not.

**Figure 2:**
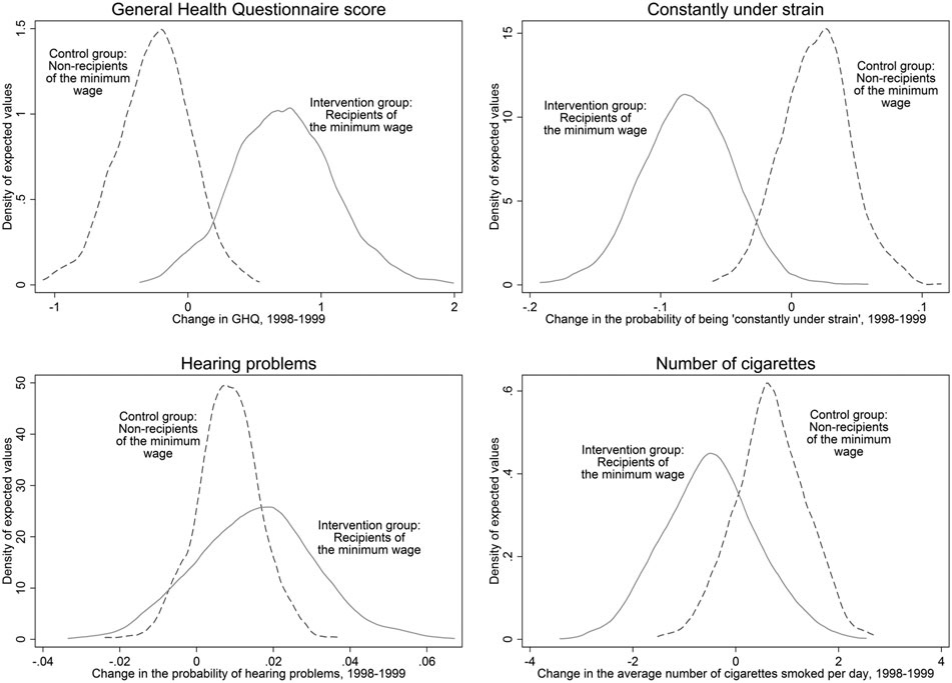
The estimated effect of the National Minimum Wage on health outcomes, control group 1, 1998–1999

The difference‐in‐differences in total GHQ scores were statistically significant (ΔGHQ score 0.93, *p* = 0.025). This improvement represents approximately 0.373 of a standard deviation, comparable in magnitude to the effect size estimated for antidepressants (0.39 of a standard deviation) on depressive symptoms (Moncrieff *et al*., 2004). Similarly, among the intervention group, there were lower probabilities of unhappiness or depression (−0.14, *p* = 0.045), being constantly under strain (0.098, *p* = 0.013), and anxiety or depression (−0.10, *p* = 0.016). There was no difference between the intervention group and control group 1 (eligible non‐recipients) in terms of the change in the likelihood of hearing problems (*p* = 0.64), experiencing elevate blood pressure (*p* = 0.58), and the number of cigarettes smoked (*p* = 0.26) (Table 2 and Figure 2).

**Table 2:** Difference‐in‐differences estimate of the health effects of the National Minimum Wage, control group 1, 1998–1999

	Equation 2: intervention effect on those receiving a wage increase (std. dev.)	Equation 3: intervention effect on those not receiving a wage increase (std. dev.)	Equation 4: difference in means: intervention‐control (standard error)	*p* value
	*n* = 63	*n* = 107	*n* = 170	
Change in mental health				
GHQ score (1998–1999)	0.70 (2.95)	−0.23 (2.97)	0.93^b^ (0.47)	0.025
‘More unhappiness or depression’^a^	−0.13 (0.52)	0.0093 (0.47)	−0.14^b^ (0.080)	0.045
‘Constantly under strain’^a^	−0.079 (0.27)	0.019 (0.27)	−0.098^b^ (0.043)	0.013
Self‐report anxiety/depression^a^	−0.063 (0.30)	0.037 (0.28)	−0.10^b^ (0.047)	0.016
Change in health behaviours				
Number of cigarettes^a^, ^b^	−0.52 (5.11)	0.19 (4.06)	0.70 (1.11)	0.26
Change in physical health problems				
Self‐report hearing problems^a^	0.016 (0.13)	0.0093 (0.097)	0.0065 (0.018)	0.64
Blood pressure	0.016 (0.22)	0.0093 (0.22)	0.0065 (0.035)	0.58

One‐tailed *t*‐test reported for difference between mean differences, based on unequal variances. Higher GHQ score captures better health (1 = worst, 13 = best).

a Box 1 describes variable coding.

b *n* = 73, because of non‐response and constraining sample to those who are smokers.

Similar results were observed using the second control group—ineligible non‐recipients (control group 2) (Web Appendix 1). Following the introduction of the minimum wage, the intervention group had significantly greater improvements in their psychiatric caseness than the control group (ΔGHQ = 1.06, *p* = 0.021) (refer to Table 3). Similarly, among the intervention group, the probability of reporting depressive symptoms declined (0.15, *p* = 0.043), whereas there was no statistically significant change in either the likelihood of experiencing hearing problems (*p* = 0.33), being diagnosed with elevated blood pressure (*p* = 0.063), or change in the number of cigarettes respondents consumed (*p* = 0.32).

**Table 3:** Difference‐in‐differences estimate of the health effects of the National Minimum Wage, control group 2, 1998–1999

	Equation 2: intervention effect on those receiving a wage increase (std. dev.)	Equation 3: intervention effect on those not receiving a wage increase (std. dev.)	Equation 4: difference in means: intervention‐control (standard error)	*p* value
	*n* = 63	*n* = 109	*n* = 172	
Change in mental health				
GHQ score (1998–1999)	0.70 (2.95)	−0.36 (3.75)	1.06^b^ (0.52)	0.021
‘More unhappiness or depression’^a^	−0.13 (0.52)	0.018 (0.54)	−0.15^b^ (0.084)	0.043
‘Constantly under strain’^a^	−0.079 (0.27)	0.018 (0.30)	−0.098^b^ (0.045)	0.016
Self‐report anxiety/depression^a^	−0.063 (0.30)	0.00 (0.30)	−0.063 (0.048)	0.095
Change in health behaviours				
Number of cigarettes^a^, ^b^	−0.52 (5.11)	0.00 (4.18)	−0.52 (1.09)	0.32
Change in physical health problems				
Self‐report hearing problems^a^	0.016 (0.13)	0.028 (0.21)	−0.012 (0.026)	0.33
Blood pressure	0.016 (0.22)	0.073 (0.26)	−0.057 (0.037)	0.063

One‐tailed *t*‐test reported for difference between mean differences, based on unequal variances. Higher GHQ score captures better health (1 = worst, 13 = best).

a Box 1 describes variable coding.

b *n* = 73, because of non‐response and constraining sample to those who are smokers.

To account for potential unobserved confounding, we regress change in GHQ on a dummy for the intervention group (=1) and a series of potential socio‐demographic differences, including age, sex, tenure, social class, education and marital status age, gender, and education. As shown in Table 4 panel A (control group 1) and panel B (control group 2), the estimated effect of the wage increase on recipients remains statistically significant, reflecting a divergence in depressive symptoms between those who receive the wage increase and those who do not.

**Table 4:** Association between receiving the minimum wage and health in control group 1 (A) and control group 2 (B), 1998–1999, adjusted for socio‐demographic controls

	ΔGHQ score between 1998 and 1999	
A. Control group 1	(1)	(2)
Intervention effect on those receiving a wage increase, NMW (yes = 1)	0.93* (0.47)	1.04* (0.49)
Adjusted for covariates	N	Y
		
Number of observations	170	166
*R* ^2^	0.023	0.15

	ΔGHQ score between 1998 and 1999	
B. Control group 2	(1)	(2)
Intervention effect on those receiving a wage increase, NMW (yes = 1)	1.06* (0.52)	1.59** (0.54)
Adjusted for covariates	N	Y
		
Number of observations	172	170
*R* ^2^	0.021	0.15

Control group 1: comparison of the eligible recipients and ineligible non‐recipients. Control group 2: comparison of the eligible recipients and eligible non‐recipients. Higher GHQ score captures better health (1 = worst, 13 = best). Observations combined from both 1998 and 1999 hence larger number of observations. Control variables include age, tenure, number of hours worked, occupational class (NS‐SEC), education, and marital status.

* *p* < 0.05, two‐tailed test.

** *p* < 0.01, two‐tailed test.

### 3.2 Potential role of financial strain

First, we tested whether receiving the minimum wage was associated with lower levels of current financial strain between 1998 and 1999. We found that the intervention group experiences less financial strain than both control group 1, eligible non‐recipients (*β* = 0.29; *p* = 0.034), and control group 2, ineligible non‐recipients (*β* = 0.35; *p* = 0.008) (Table 5). By regressing change in GHQ on change in an individual's current financial situation, we found that improvement in respondents' financial circumstances was associated with better psychiatric caseness score in both control groups 1 (*β* = 0.76; *p* = 0.012) and 2 (*β* = 1.13; *p* = 0.0014).

**Table 5:** Association between changes in financial strain and receiving the minimum wage in control group 1 and control group 2, 1998–1999

Covariates	ΔFinancial strain score between 1998 and 1999	
	Intervention and control group 1	Intervention and control group 2
	(1)	(2)
Intervention effect on those receiving a wage increase, NMW (yes = 1)	0.29* (0.13)	0.35** (0.13)
	
		
Number of observations	170	172
*R* ^2^	0.025	0.040

Constant estimated in the model (not shown). Control group 1: comparison of the eligible recipients and ineligible non‐recipients. Control group 2: comparison of the eligible recipients and eligible non‐recipients. Higher financial scores capture greater financial security (1 = finding it very difficult, 5 = living comfortably). Box 1 defines financial strain.

* *p* < 0.05, two‐tailed test.

** *p* < 0.01, two‐tailed test.

Next, we tested whether financial strain mediated the effect of the minimum wage on mental health, or psychiatric caseness, by regressing change in GHQ on minimum wage indicator and changes in financial strain. We observed that adjusting for financial strain attenuated the effect of receiving the National Minimum Wage in both control groups 1 (*p* = 0.11) and 2 (*p* = 0.18) (Table 6).

**Table 6:** Mediating effect of financial strain on the minimum wage‐health association in control group 1 (A) and control group 2 (B), 1998–1999

	ΔGHQ score between 1998 and 1999	
A. Control group 1	(1)	(2)
Intervention effect on those receiving a wage increase, NMW (yes = 1)	0.93* (0.47)	0.73 (0.46)
Change in financial strain	—	0.69* (0.30)
Number of observations	170	170
*R* ^2^	0.023	0.062
	

	ΔGHQ score between 1998 and 1999	
B. Control group 2	(1)	(2)
Intervention effect on those receiving a wage increase, NMW (yes = 1)	1.06* (0.52)	0.69 (0.51)
Change in financial strain	—	1.06** (0.36)
Number of observations	172	172
*R* ^2^	0.021	0.083

Constant estimated in the model (not shown). Control group 1: comparison of the eligible recipients and ineligible non‐recipients. Control group 2: comparison of the eligible recipients and eligible non‐recipients. Higher GHQ score captures better health (1 = worst, 13 = best). Higher financial scores captures greater financial security (1 = finding it very difficult, 5 = living comfortably). Box 1 defines financial strain.

* *p* < 0.05, two‐tailed test.

** *p* < 0.01, two‐tailed test.

We assessed whether the positive mental health effects were sustained after the initial year of wage increase by adding a subsequent year, 2000/2001. We observed that between 1999/2000 and 2000/2001, there was no significant change in the mean GHQ score in the intervention group, indicating that the improvement persisted. In parallel, mean GHQ scores did not significantly change in either control group in this same period.

### 3.3 Robustness tests

First, we compared estimates from the natural experiment to those from multivariable regression models (refer to Web Appendix 2). Web Appendix 2 reports a series of regression models, where each coefficient in each cell is from a separate model and each row is a different outcome variable. We estimate a series of random‐effects linear regression models (controlling for time dummies) that include observations from 1994 to 2001 (before and after the intervention in 1999) in order to estimate the health effects of a £1 increase in hourly wages across this period. To capture any non‐linearity in the income‐health association, we use a sample of low‐wage workers (<£10), divided into those below the median (column 1 = those earning below £6 per hour in the previous year (*n* = 4478)) and those above the median in this sub‐sample (column 2 = earning between £6 and £10 per hour in the previous year (*n* = 4411)). We estimated that each £1 increase in wage was associated with significant increases in GHQ scores of 0.038 (*p* = 0.043) and lower probabilities of depression (−0.011, *p* < 0.001) for those on low wages, that is, <£6 per hour (Web Appendix 2). In contrast, there was no clear association between increasing wages and the change in GHQ score among persons whose earnings were above the median in the low‐wage sub‐sample (ΔGHQ score 0.0072, *p* = 0.65).

Control group 1 was defined as those who earn between 100 and 110% of the minimum wage. To test whether restricting the post‐intervention wages for the treatment group influences our results, we increase the upper limit incrementally by 10 pence (£0.10) in a series of models (Web Appendix 3). The original difference‐in‐difference estimate is 1.01 (*p* = 0.037). If the control group includes those who earn up to £4.10, then the difference‐in‐difference estimate is 1.17 (*p* = 0.013). If the control group includes those who earn up to £4.20, then the difference‐in‐difference estimate is 0.93 (*p* = 0.044). If the control group includes those who earn up to £4.30, then the difference‐in‐difference estimate is 0.83 (*p* = 0.064). And finally, if the control group includes those who earn up to £4.40, then the difference‐in‐difference estimate is 0.81 (*p* = 0.07). The effect is clearly attenuated slightly as earnings increase, but the magnitude of the effect on mental health is large and is close to being significant, even at ~20% above the minimum wage threshold.

One possible source of confounding is from second jobs; however, in the analytic sample, the numbers reporting multiple jobs were small (<5% of the sample). None received the minimum wage. Hence, when excluding this sub‐set with second jobs, our results were unchanged (Web Appendix 4). Another source of confounding concerns those who might be earning below the minimum wage because of an exemption, such as those who receive accommodation from their employer. We remove these individuals (*n* = 7) from our models to test whether they influence our results and find that they do not (ΔGHQ = 1.05, *p* = 0.050). Additionally, we assessed job satisfaction over time to test whether differences between employers are likely to explain the psychological benefits we observe for the intervention group. We find that the intervention group experienced greater but non‐significant declines in job satisfaction between 1998 and 1999 (difference‐in‐difference = 0.066, *p* = 0.81, *n* = 172) (Web Appendix 5). This is somewhat unexpected given that increased wages can lead to greater job satisfaction (Lydon and Chevalier, 2002). However, this wage increase is a response to a government‐mandated policy and not a reflection of managerial generosity, so the influence of an increase in wages on job satisfaction may vary depending on the source of the increase, e.g. employer or government.

Selecting the intervention group based on post‐intervention wages may generate bias if those who remain below the minimum wage are more susceptible to worsening mental health. This is especially relevant to control group 2, including persons whom were eligible but did not receive the wage uplift. To test this possibility, we re‐estimate the difference‐in‐difference models of changes in GHQ on membership in each control group between 1998 and 1999. If selection effects were substantial, it would mean that control groups 1 and 2 did not have parallel trajectories; however, we did not detect this pattern (difference‐in‐difference estimate between control group 1 and control group 2 = 0.026, *p* = 0.88). In fact, control group 1 and control group 2 are similar in terms of their GHQ scores before the intervention (*p* = 0.74), with no statistically discernable difference between them over time (*p* = 0.41).

Consistent with prior research, we found no effect of the introduction of the minimum wage on the likelihood of employment for either intervention or control groups between 1998 and 1999 (Web Appendix 6). To reduce the positive skew in GHQ‐12, we re‐estimate our models using the natural log of the dependent variable, finding that our results do not qualitatively change (*p* = 0.018). Comparing the intervention group with all other people in the sample over the age of 21, we find that the effect is attenuated slightly but remains largely distinct (ΔGHQ score 0.67, *p* = 0.089, *n* = 8585). Currently in this paper, we assume that, on average over the month, overtime payments are similar to the contracted hourly wage, and so, the overtime premium is relatively small. Yet, this is almost certainly an underestimate. As a robustness check, we now calculate an overtime premium of 25, 50, and 75%. We report our difference‐in‐differences for both control groups—showing that none of the results was changed with this adjustment (refer to Web Appendix 7). Combining both control groups (control group *n* = 216; intervention group *n* = 63), we observe that the introduction of the minimum wage improved mental health among the intervention group over and above any change in the combined control group (ΔGHQ score for intervention group: 0.99, *p* = 0.023). Following Stewart (2004a), we re‐estimate our models using a wage gap estimator (refer to equation 2 on page 72) that captures the change in income because of the minimum wage using the gap between the minimum wage threshold in 1999 (£3.60) and actual wage in 1998. For example, if person *A* earned £3.20 per hour in 1998, then the wage gap would be 0.40, while for person *B*—who is in the control group—the wage gap would be 0. Using this approach, we find that our results are attenuated slightly but are of similar magnitude to our previous estimates and are in the same direction (Web Appendix 8).

Finally, our approach to defining the intervention group in this paper differs from previous research because we condition on post‐intervention wages. To relax this assumption, we keep the same control groups but re‐estimate our models by including all those individuals who were earning less than £3.60 in 1998 and then were earning more than £3.60 per hour in 1999. Again, the results are in the same direction, albeit attenuated (Web Appendix 9). This likely reflects that wage increases this large are not because of minimum wage but other factors unrelated to the policy. For example, if we compare this new intervention group with the control group 2, in contrast to the main results, we now find that the intervention group are more likely to be male, are younger, earn more money, work more hours, and less likely to have a full‐time job than the control group.

## 4 Discussion

Our study uses a natural experiment design to approximate an ‘as‐if random’ controlled trial of wage increases *in milieu* among low‐paid workers. Using two control groups that fulfil as‐if randomisation criteria prior to the intervention, we were able to identify important differences in groups exposed to wage rises because of the National Minimum Wage intervention. This approach advances existing scholarship by addressing some of Bradford‐Hill's criteria for a causal relationship that was previously missing from the literature, including specificity (low‐wage workers), strength of association (comparable with the effect of antidepressants), plausibility, consistency, temporal relationship (divergence follows policy intervention), and, partially, experimental evidence (Bradford‐Hill, 1965). The authors of earlier reviews of the health effects of increases in wages, which included income maintenance experiments, financial aid to ex‐prisoners, and lottery winners, noted how opportunities to evaluate the effects of natural experiments had often been missed (Connor *et al*., 1999).

By adding to this sparse literature, this study makes three main contributions to knowledge. First, the introduction of a National Minimum Wage improved reported mental health among low‐paid people, reducing their probability of anxiety and depression. Second, it shows that these health benefits are mediated, at least in part, through changes in the financial strain that low‐paid people experience, with effects that are sustained over time. Third, while it has been argued that wage rises in low‐wage workers may be deleterious for health if additional funds are used to consume tobacco, we found no such evidence of increased tobacco use. Consistent with previous work that observes that tobacco is an inferior good, we observed a slightly negative albeit not statistically significant relationship (*p* > 0.05) between increased wages and tobacco in this UK minimum wage experiment (Chaloupka and Warner, 2000).

Notwithstanding the strength of the study methodology, our analysis has several limitations. First, like many natural experiments, the study's sample size is small because, even with the large sample in the BHPS, the introduction of the National Minimum Wage only influenced the lives of a minority of the employed (Dickens and Manning 2004a; Dickens and Manning 2004b). Despite this smaller sample, which would weaken the study's statistical power, the analysis was able to detect strong, statistically significant effects of the National Minimum Wage. The observed change of over one point on the 12‐point scale (0.3 of a standard deviation) is comparable with the effect of antidepressants on depression and is not trivial (Moncrieff *et al*., 2004). By more precisely isolating intervention effects, the natural experimental design was able to identify a larger effect size, overcoming problems associated with traditional regression‐based estimates, despite having fewer observations.

Second, unlike with RCTs, it was not possible to eliminate all sources of potential selection bias between intervention and control groups. For example, it is not possible to identify all of those persons who might be exempt from the minimum wage, such as those who are receiving a training rate or whose wage is reduced because they receive accommodation from their employer. Nevertheless, because these groups likely form a very small number of people in our sample, they are unlikely to substantial influence our results, although more research will be needed to examine how the minimum wage may have influenced these groups. Further, prior to the wage intervention, both study and control groups were statistically similar with respect to socio‐demographic factors and the health outcomes of interest. Particularly in the non‐compliance control group, there may have been unobserved employer characteristics that led mental health to deteriorate. However, there was no pre‐intervention difference in job satisfaction between control and intervention group on workplace characteristics. Further, our study also found significant improvements in mental health among the intervention group but no significant change among the control groups. The study also had a high degree of specificity, observing no significant effect on physical outcomes such as hearing loss that would not plausibly change in a short period of time, given the absence of a plausible biological mechanism. Taken together, these findings strongly indicate that the association is not because of an unobserved selection bias among the control groups.

Third, the measures of physical health are self‐reported and therefore may under‐report any change in prevalence of elevated blood pressure because of a change in financial circumstances. Null effects should be interpreted with some caution for this indicator. Fourth, introducing a minimum wage may also increase wages for those who are already just above the minimum wage threshold, and so, those who are untreated could also be influenced by the intervention (Morgan and Winship, 2007). While such spillover effects are theoretically plausible, and this appears to have occurred in the USA, previous studies suggest that they did not influence the UK's wage distribution (Dickens and Manning 2004a; Dickens and Manning 2004b; Stewart, 2012). Fifth, while there was no attrition in our analytic sample during the study period, there may have been attrition among these groups before 1998. However, because attrition rates are so low in the BHPS, it is unlikely that this would have substantially influenced our results. Finally, the study findings were consistent even after adjusting for a range of potentially confounding socio‐demographic factors and workplace characteristics such as job satisfaction. Hence, unobserved confounding in the non‐compliance sample is unlikely to account for the study's findings, adding support to the study's ability to draw causal inference.

Importantly, the study suggests that increasing wages does not lead to unintended adverse outcomes among persons who did not receive wage increases and further identified that financial strain was an important factor influencing the relationship between wage interventions and depressive symptoms. These findings are also consistent with recent randomised controlled trials indicating that conditions of poverty increase psychological pressure, which can impede decision‐making and resultant welfare outcomes (Mani *et al*., 2013). Additionally, while it is possible that persons who receive wage increase experience only short‐term benefits, subsequently regressing to previous levels of welfare, we found that the reduction in depressive symptoms was sustained at least up to 18–22 months after intervention. Future research is needed to investigate the longer term effects of wage increases on the health and well‐being of low‐wage groups.

Further research is also needed to evaluate the health effects of alternative mechanisms of income intervention, such as the UK's earned income tax credits, as well as to understand the effects of larger income gains among low‐paid workers, currently being debated in the USA and UK. Overall, our results indicate that increasing wages is likely to improve mental health, for example, by reducing depression and by alleviating financial strain among low‐paid workers.

## Funding

This study is part of the DEMETRIQ project. This study was carried out with financial support from the Commission of the European Communities, Grant Agreement No. 278511. The study does not necessarily reflect the commission's views and in no way anticipates the commission's future policy in this area. DS is also funded by a Wellcome Trust Investigator Award.

## Conflict of Interest

We declare that we have no conflicts of interest.

## Authorship

AR DS designed the research; AR DS performed the research, wrote the first draft of the paper, and analysed the data; AR DS JM MM MW contributed to the interpretation of the data and writing of the manuscript.

## Supporting information

Supporting info item

## References

[hec3336-bib-0001] Adebowale T , Adelufosi A . 2013 Stress and minor psychiatric morbidity among nigerian executives: some socio‐demographic and biological correlates. Annals of medical and health sciences research 3(3): 412–416. 24116324 10.4103/2141-9248.117946 PMC3793450

[hec3336-bib-0002] Angrist JD , Pischke J‐S . 2009 Mostly harmless econometrics: an empiricist's companion, Princeton University Press: Princeton, N.J.

[hec3336-bib-0003] Apouey B , Clark AE . 2015 Winning big but feeling no better? The effect of lottery prizes on physical and mental health. Health Economics 24(5): 516–538. 24677260 10.1002/hec.3035 PMC4180795

[hec3336-bib-0004] Argyle M . 1987 The psychology of happiness, Methuen: London.

[hec3336-bib-0005] Averett S , Wang Y . 2013 The effects of earned income tax credit payment expansion on maternal smoking. Health Economics 22(11): 1344–1359. 23239400 10.1002/hec.2886

[hec3336-bib-0006] Banks MH . 1983 Validation of the general health questionnaire in a young community sample. Psychological Medicine 13(2): 349–353. 6878521 10.1017/s0033291700050972

[hec3336-bib-0007] Bentley R , Baker E , Mason K , Subramanian SV , Kavanagh AM . 2011 Association between housing affordability and mental health: a longitudinal analysis of a nationally representative household survey in australia. American Journal of Epidemiology 174(7): 753–760. 21821543 10.1093/aje/kwr161

[hec3336-bib-0008] Bentley R , Pevalin D , Baker E , Mason K , Reeves A , Beer A . 2015 Housing affordability, tenure and mental health in australia and the united kingdom: a comparative panel analysis. Housing Studies.

[hec3336-bib-0009] Benzeval M , Judge K . 2001 Income and health: the time dimension. Social Science & Medicine 52(9): 1371–1190. 11286362 10.1016/s0277-9536(00)00244-6

[hec3336-bib-0010] Bertotti M , Watts P , Netuveli G , Yu G , Schmidt E , Tobi P , Lais S , Renton A . 2013 Types of social capital and mental disorder in deprived urban areas: a multilevel study of 40 disadvantaged london neighbourhoods. PLoS One 8(12): e80127. 10.1371/journal.pone.0080127 PMC3846561 24312459

[hec3336-bib-0011] Bianchini V , Roncone R , Tomassini A , Necozione S , Cifone MG , Casacchia M , Pollice R . 2013 Cognitive behavioral therapy for young people after l'aquila earthquake. Clinical practice and epidemiology in mental health : CP & EMH 9: 238–242. 24358053 10.2174/1745017901309010238 PMC3866707

[hec3336-bib-0012] Blanchflower DG , Oswald AJ . 2008 Hypertension and happiness across nations. Journal of Health Economics 27(2): 218–233. 18199513 10.1016/j.jhealeco.2007.06.002

[hec3336-bib-0013] Bor J , Basu S , Coutts , McKee M , Stuckler D . 2013 Alcohol use during the great recession of 2008‐2009. Alcohol and Alcoholism 48(3): 343–348. 23360873 10.1093/alcalc/agt002

[hec3336-bib-0014] Bradford‐Hill A . 1965 The environment and diseases: association or causation? Proceedings of the Royal Society of Medicine 58: 295–300. 14283879 10.1177/003591576505800503 PMC1898525

[hec3336-bib-0015] Chaloupka FJ , Straif K , Leon ME . 2011 Effectiveness of tax and price policies in tobacco control. Tobacco Control 20(3): 235–238. 21115556 10.1136/tc.2010.039982

[hec3336-bib-0016] Chaloupka FJ , Warner KE . 2000 The economics of smoking 1539‐627 in Handbook of health economics, edited by Pauly MV, TG McGuire , Barros PP.

[hec3336-bib-0017] Clarke K , Saville N , Shrestha B , Costello A , King M , Manandhar D , Osrin D , Prost A . 2013 Predictors of psychological distress among postnatal mothers in rural nepal: a cross‐sectional community‐based study. Journal of Affective Disorders. 10.1016/j.jad.2013.11.018 PMC3969296 24370265

[hec3336-bib-0018] Connor J , Rodgers A , Priest P . 1999 Randomised studies of income supplementation: a lost opportunity to assess health outcomes. Journal of Epidemiology & Community Health 53(11): 725–730. 10656103 10.1136/jech.53.11.725 PMC1756807

[hec3336-bib-0019] Costello EJ , Compton SN , Keeler G , Angold A . 2003 Relationships between poverty and psychopathology: a natural experiment. Journal of the American Medical Association 290(15): 2023–2029. 14559956 10.1001/jama.290.15.2023

[hec3336-bib-0020] Craig P , Cooper C , Gunnell D , Haw S , Lawson K , Macintyre S , Ogilvie D , Petticrew M , Reeves B , Sutton M , Thompson S . 2012 Using natural experiments to evaluate population health interventions: new medical research council guidance. Journal of Epidemiology & Community Health 66(12): 1182–1186. 22577181 10.1136/jech-2011-200375 PMC3796763

[hec3336-bib-0021] Deaton A . 2003 Health, inequality, and economic development. Journal of Economic Literature 41(1): 113–158.

[hec3336-bib-0022] Dickens R , Manning A . 2004 Has the national minimum wage reduced uk wage inequality? Journal of the Royal Statistical Society Series a‐Statistics in Society 167: 613–626.

[hec3336-bib-0023] Dickens R . 2004 Spikes and spill‐overs: the impact of the national minimum wage on the wage distribution in a low‐wage sector. Economic Journal 114(494): C95–C101.

[hec3336-bib-0024] Dunning T . 2008 Improving causal inference—strengths and limitations of natural experiments. Political Research Quarterly 61(2): 282–293.

[hec3336-bib-0025] Dunning T . 2012 Natural experiments in the social sciences, Cambridge University Press: Cambridge.

[hec3336-bib-0026] Frijters P , Ulker A . 2008 Robustness in health research: do differences in health measures, techniques, and time frame matter? Journal of Health Economics 27(6): 1626–1644. 18639357 10.1016/j.jhealeco.2008.06.003

[hec3336-bib-0027] Goldberg DP , Gater R , Sartorius N , Ustun TB , Piccinelli M , Gureje O , Rutter C . 1997 The validity of two versions of the ghq in the who study of mental illness in general health care. Psychological Medicine 27(1): 191–197. 9122299 10.1017/s0033291796004242

[hec3336-bib-0028] Goldberg DP . 1978 Manual of the general health questionnaire, NFER: Windsor.

[hec3336-bib-0029] Gunasekara FI , Carter K , Blakely T . 2011 Change in income and change in self‐rated health: systematic review of studies using repeated measures to control for confounding bias. Social Science and Medicine 72(2): 193–201. 21146277 10.1016/j.socscimed.2010.10.029

[hec3336-bib-0030] Heckman JJ . 2008 Econometric causality. International Statistical Review 76(1): 1–27.

[hec3336-bib-0031] Hoynes H , Miller D , Simon D . 2015 Income, the earned income tax credit, and infant health. American Economic Journal‐Economic Policy 7(1): 172–211.

[hec3336-bib-0032] Ipsos MORI . 2012 Non‐compliance with the national minimum wage, Low Pay Commission (ed.), Low Pay commission: London.

[hec3336-bib-0033] Jenkins S. 2010 The british household panel survey and its income data. ISER Working Paper Series (2010‐33).

[hec3336-bib-0034] Jensen R , Miller N . 2008 Giffen behaviour and subsistence consumption. American Economic Review 98(4): 1553–1577. 21031158 10.1257/aer.98.4.1553 PMC2964162

[hec3336-bib-0035] Jetter KM , Cassady DL . 2006 The availability and cost of healthier food alternatives. American Journal of Preventive Medicine 30(1): 38–44. 16414422 10.1016/j.amepre.2005.08.039

[hec3336-bib-0036] Jones AM , Wildman J . 2008 Health, income and relative deprivation: evidence from the bhps. Journal of Health Economics 27(2): 308–324. 18207266 10.1016/j.jhealeco.2007.05.007

[hec3336-bib-0037] Kim D , Leigh JP . 2010 Estimating the effects of wages on obesity. Journal of Occupational and Environmental Medicine 52(5): 495–500. 20431413 10.1097/JOM.0b013e3181dbc867

[hec3336-bib-0038] King G , T M , Wittenberg J . 2000 Making the most of statistical analyses: improving interpretation and presentation. American Journal of Political Science 44: 341–355.

[hec3336-bib-0039] Krieger N , Williams DR , Moss NE . 1997 Measuring social class in us public health research: concepts, methodologies, and guidelines. Annual Review of Public Health 18: 341–378. 10.1146/annurev.publhealth.18.1.341 9143723

[hec3336-bib-0040] Leigh A . 2007 Earned income tax credits and labor supply: new evidence from a british natural experiment. National Tax Journal 60(2): 205–224.

[hec3336-bib-0041] Leigh JP , Du J . 2012 Are low wages risk factors for hypertension? European Journal of Public Health 22(6): 854–859. 22262559 10.1093/eurpub/ckr204 PMC3598375

[hec3336-bib-0042] Lorant V , ge DeliÃ D , Eaton W , Robert A , Philippot P , Ansseau M . 2003 Socioeconomic inequalities in depression: a meta‐analysis. American Journal of Epidemiology 157(2): 98–112. 12522017 10.1093/aje/kwf182

[hec3336-bib-0043] Low Pay Commission . 2000 The national minimum wage: the story so far. in Second Report of the Low Pay Commission London: Low Pay Commission.

[hec3336-bib-0044] Lydon R , Chevalier A . 2002 Estimates of the effect of wages on job satisfaction. CEPDP 531: .

[hec3336-bib-0045] Lynn P , Buck N , Burton J , Laurie H , Noah Uhrig SC . 2006 In Quality profile: British household panel survey: waves 1 to 13: 1991‐2003, Lynn P (ed.), ISER: Colchester, England.

[hec3336-bib-0046] Mackenbach JP , Martikainen P , Looman CWN , Dalstra JAA , Kunst AE , Lahelma E , Breeze E , Cambois E , Grundy E , Lunde E , van Oyen H , Rasmussen N . 2005 The shape of the relationship between income and self‐assessed health: an international study. International Journal of Epidemiology 34(2): 286–293. 15561750 10.1093/ije/dyh338

[hec3336-bib-0047] Mani A , Mullainathan S , Shafir E , Zhao J . 2013 Poverty impedes cognitive function. Science 341(6149): 976–980. 23990553 10.1126/science.1238041

[hec3336-bib-0048] Marmot M . 2002 The influence of income on health: views of an epidemiologist. Health Aff (Millwood) 21(2): 31–46. 10.1377/hlthaff.21.2.31 11900185

[hec3336-bib-0049] McCabe CJ , Thomas KJ , Brazier JE , Coleman P . 1996 Measuring the mental health status of a population: a comparison of the ghq‐12 and the sf‐36 (mhi‐5). British Journal of Psychiatry 169(4): 516–521. 8894205 10.1192/bjp.169.4.516

[hec3336-bib-0050] McCarrier KP , Zimmerman FJ , Ralston JD , Martin DP . 2011 Associations between minimum wage policy and access to health care: evidence from the behavioral risk factor surveillance system, 1996‐2007. American Journal of Public Health 101(2): 359–367. 21164102 10.2105/AJPH.2006.108928 PMC3020211

[hec3336-bib-0051] Moncrieff J , Wessely S , Hardy R . 2004 Active placebos versus antidepressants for depression. Cochrane Database of Systematic Reviews 1: 1–31. 10.1002/14651858.CD003012.pub2 PMC8407353 14974002

[hec3336-bib-0052] Morgan SL , Winship C . 2007 Counterfactuals and causal inference: methods and principles for social research, Cambridge University Press: Cambridge.

[hec3336-bib-0053] Neumark D , Schweitzer M , Wascher W . 2004 Minimum wage effects throughout the wage distribution. Journal of Human Resources 39(2): 425–450.

[hec3336-bib-0054] Neumark D , Wascher W . 2001 Using the eitc to help poor families: new evidence and a comparison with the minimum wage. National Tax Journal 54(2): 281–317.

[hec3336-bib-0055] Pearl J . 1995 Causal diagrams for empirical research. Biometrika 82(4): 669–688.

[hec3336-bib-0056] Pearl J . 2010 The foundations of causal inference. Sociological Methodology 40(40): 75–149.

[hec3336-bib-0057] Pearl J . 2000 Causality: models, reasoning, and inference, Cambridge University Press: Cambridge.

[hec3336-bib-0058] Petticrew M , Cummins S , Ferrell C , Findlay A , Higgins C , Hoy C , Kearns A , Sparks L . 2005 Natural experiments: an underused tool for public health? Public Health 119(9): 751–757. 15913681 10.1016/j.puhe.2004.11.008

[hec3336-bib-0059] Petticrew M , McKee M , Lock K , Green J , Phillips G . 2013 In search of social equipoise. BMJ 347: f4016. 23828896 10.1136/bmj.f4016

[hec3336-bib-0060] Pevalin DJ . 2000 Multiple applications of the ghq‐12 in a general population sample: an investigation of long‐term retest effects. Social Psychiatry and Psychiatric Epidemiology 35(11): 508–512. 11197926 10.1007/s001270050272

[hec3336-bib-0061] Reang T , Bhattacharjya H . 2013 A study to assess the emotional disorders with special reference to stress of medical students of agartala government medical college and govinda ballabh pant hospital. Indian journal of community medicine : official publication of Indian Association of Preventive & Social Medicine 38(4): 207–211. 24302820 10.4103/0970-0218.120154 PMC3831689

[hec3336-bib-0076] Reeves A , McKee M , Clair A , Stuckler D . 2016 Reductions in housing benefit increases symptoms of depression in low‐income UK households. American Journal of Epidemiology. 10.1093/aje/kww055 PMC5023793 27613659

[hec3336-bib-0062] Risberg T , Jacobsen BK . 2003 The association between mental distress and the use of alternative medicine among cancer patients in north norway. Quality of life research : an international journal of quality of life aspects of treatment, care and rehabilitation 12(5): 539–544. 10.1023/a:1025063705413 13677498

[hec3336-bib-0063] Robinson G , McNulty JE , Krasno JS . 2009 Observing the counterfactual? The search for political experiments in nature. Political Analysis 17(4): 341–357.

[hec3336-bib-0064] Sacker A , Wiggins R , Bartley M , McDonough P . 2007 Self‐rated health trajectories in the united states and the united kingdom: a comparative study. American Journal of Public Health 97(5): 812–818. 17395850 10.2105/AJPH.2006.092320 PMC1854880

[hec3336-bib-0065] Sekhon JS , Titiunik R . 2012 When natural experiments are neither natural nor experiments. American Political Science Review 106(1): 35–57.

[hec3336-bib-0066] Skapinakis P , Weich S , Lewis G , Singleton N , Araya R . 2006 Socio‐economic position and common mental disorders: longitudinal study in the general population in the uk. British Journal of Psychiatry 189(AUG.): 109–117. 16880479 10.1192/bjp.bp.105.014449

[hec3336-bib-0067] Smith JP . 1999 Healthy bodies and thick wallets: the dual relation between health and economic status. The Journal of Economic Perspectives 13(2): 144–166. 15179962 PMC3697076

[hec3336-bib-0068] Stewart MB . 2012 Wage inequality, minimum wage effects, and spillovers. Oxford Economic Papers‐New Series 64(4): 616–634.

[hec3336-bib-0069] Stewart MB , Swaffield JK . 2002 Using the bhps wave 9 additional questions to evaluate the impact of the national minimum wage. Oxford Bulletin of Economics and Statistics 64(SUPPL.): 633–652.

[hec3336-bib-0070] Stewart MB . 2004a The impact of the introduction of the uk minimum wage on the employment probabilities of low‐wage workers. Journal of the European Economic Association 67–97.

[hec3336-bib-0071] Stewart MB . 2004b The employment effects of the national minimum wage. Economic Journal 114(494): C110–C116.

[hec3336-bib-0072] Taylor MP , Jenkins SP , Sacker A . 2011 Financial capability and psychological health. Journal of Economic Psychology 32(5): 710–723.

[hec3336-bib-0073] Taylor MP , Pevalin DJ , Todd J . 2007 The psychological costs of unsustainable housing commitments. Psychological Medicine 37(7): 1027–1036. 17224094 10.1017/S0033291706009767

[hec3336-bib-0074] Theofilou P . 2011 Quality of life in patients undergoing hemodialysis or peritoneal dialysis treatment. Journal of clinical medicine research 3(3): 132–138. 21811544 10.4021/jocmr552w PMC3138410

[hec3336-bib-0075] Noah Uhrig SC . 2008 The nature and causes of attrition in the british household panel survey. ISER Working Paper Series 2008(05): 1–85.

