# Supplementary material for: Introduction of a National Minimum Wage Reduced Depressive Symptoms in Low‐Wage Workers: A Quasi‐Natural Experiment in the UK
Source: Health Econ. 2016 Apr 4;26(5):639–55. doi: 10.1002/hec.3336 (PMC5396382; doi:10.1002/hec.3336)
Supplement: Supplementary file 1 — Supporting info item [file HEC-26-639-s001.docx]

**Supporting Information**

Web Appendix 1: The estimated effect of the National Minimum Wage on health outcomes, control group 2, 1998-1999

Web Appendix 2: Unadjusted association between increased income and change in health outcomes, between 1994-2001.

Web Appendix 3: Difference-in-difference of change in GHQ score in intervention and control groups, with different restrictions on the control group, 1998-1999

Web Appendix 4: Adjusted difference-in-difference of change in GHQ score in intervention and control group 1 and control group 2, adjusted for second job, 1998-1999

Web Appendix 5: Change in job satisfaction between intervention group and control group 1 (ineligible non-recipients) and control group 2 (eligible non-recipients), 1998-1999.

Web Appendix 6: The probability of becoming unemployed between intervention group and control group 1 (ineligible non-recipients) and control group 2 (eligible non-recipients), 1998-1999.

Web Appendix 7: Difference-in-difference of change in GHQ score in intervention and control groups, adjusted for overtime premium at 25%, 50%, and 75%, 1998-1999

Web Appendix 8: Association between the wage-gap (difference between minimum wage and actual wages) for the intervention group and control group

Web Appendix 9: Association between receiving the minimum wage and health in control group 1 (A) and control group 2 (B), 1998-1999, with and without restriction on post-intervention wages

Web Appendix 1: The estimated effect of the National Minimum Wage on health outcomes, control group 2, 1998-1999


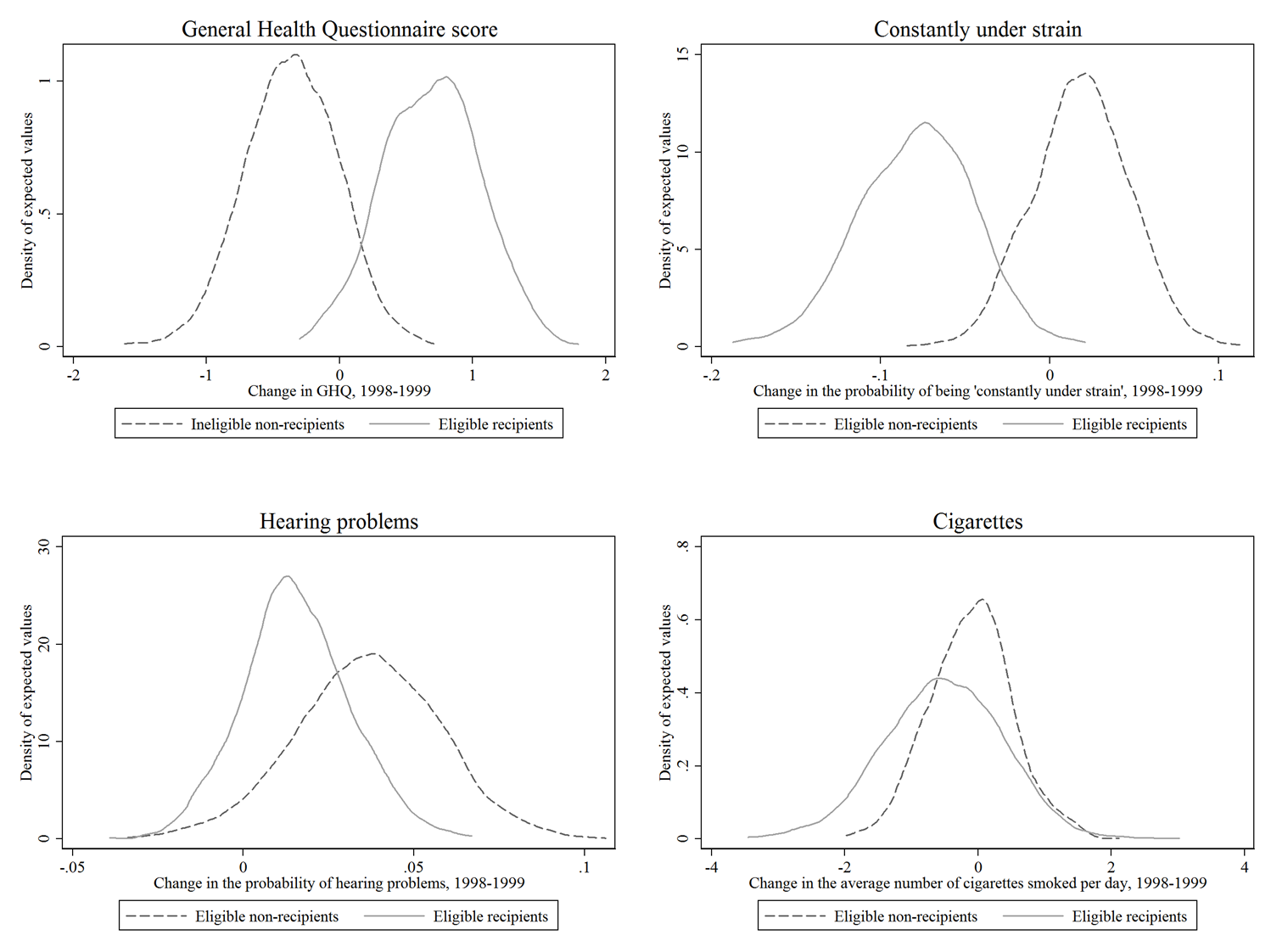


*Notes:* Higher ΔGHQ score reflect improved mental health. Density estimates are based on the difference-in-difference models reported in Table 3. Variance of the intervention and control group means were estimated from 1000 repeated draws of the sample probability distributions using STATA module CLARIFY.

Web Appendix 2: Unadjusted association between increased income and change in health outcomes, between 1994-2001.

|  | £1 increase in hourly income between t & t-1 | |
| --- | --- | --- |
| **Dependent variables** | <£6.00 per hour  (95% Confidence Interval)  (1) | £6.00-£10.00 per hour  (95% Confidence Interval)  (2) |
| *Change in mental health* |  |  |
| GHQ score (t & t-1) | 0.038*  (0.0011 to 0.75) | 0.0072  (-0.024 to 0.038) |
| ‘More unhappiness or depression’^1^ (t & t-1) | -0.011**  (-0.0046 to -0.017) | -0.0013  (-0.0063 to 0.0037) |
| ‘Constantly under strain’^2^ (t & t-1) | -0.0017  (-0.0050 to 0.0015) | -0.0014  (-0.0038 to 0.00090) |
| Self-report anxiety/depression^3^ (t & t-1) | -0.00076  (-0.0037 to 0.0022) | 0.0024*  (0.00013 to 0.0048) |
| *Change in health behaviours* |  |  |
| Number of cigarettes^4*^ (t & t-1) | -0.051  (-0.16 to 0.061) | 0.036  (-0.073 to 0.15) |
| *Change in physical health problems* |  |  |
| Self-report hearing problems^5^ (t & t-1) | -0.00090  (-0.0022 to 0.00044) | -0.00075  (-0.0022 to 0.00066) |
| Blood pressure (t & t-1) | -0.0011  (-0.0037 to 0.0014) | 0.00051  (-0.0015 to 0.0025) |
| Number of individuals | 4478 | 4411 |
| Person-years | 11322 | 11047 |

*Notes:* Confidence intervals are based on standard errors adjusted for repeated observations. Higher GHQ score captures better health (1 = worst, 13 = best). Sample is constrained to include only those respondents whose annual change in income is between -£20 per hour or +£20 per hour. This excludes 49 observations from the analytic sample. All models include time dummies.

Each coefficient comes from a separate model.

* p <0.05, ** p < 0.01

Web Appendix 3: Difference-in-difference of change in GHQ score in intervention and control groups, with different restrictions on the control group, 1998-1999

| **Control group 1 (Above)** | GHQ score between 1998-1999 | | | | |
| --- | --- | --- | --- | --- | --- |
|  | Control group  £3.60-£4.00 | Control group  £3.60-£4.10 | Control group  £3.60-£4.20 | Control group  £3.60-£4.30 | Control group  £3.60-£4.40 |
|  | (1) | (2) | (3) | (4) | (5) |
| Intervention effect on those receiving a wage increase, NMW (yes = 1) | 1.01^*^ | 1.17^*^ | 0.93^*^ | 0.83 | 0.81 |
|  | (0.48) | (0.47) | (0.46) | (0.45) | (0.44) |
| Difference between the control group baseline (the regression constant) and the intervention group in 1998 | -0.40 | -0.58 | -0.41 | -0.42 | -0.43 |
|  | (0.48) | (0.45) | (0.45) | (0.44) | (0.44) |
| Control group baseline in 1998 | 11.4^**^ | 11.5^**^ | 11.4^**^ | 11.4^**^ | 11.4^**^ |
|  | (0.25) | (0.20) | (0.20) | (0.18) | (0.17) |
| Time trend in the control group, 1998-1999 | -0.25 | -0.37 | -0.13 | -0.032 | -0.0090 |
|  | (0.29) | (0.25) | (0.23) | (0.21) | (0.21) |
|  |  |  |  |  |  |
| Observations | 331 | 413 | 475 | 544 | 615 |
| *R^2^* | 0.0091 | 0.010 | 0.0063 | 0.0052 | 0.0045 |

Notes: Constant included in the model (reported as control group baseline in 1998). Standard errors clustered by individual to correct for repeated observations (intra-individual differences). Higher GHQ score captures better health (1 = worst, 13 = best).

* p <0.05, ** p < 0.01

Web Appendix 4: Adjusted difference-in-difference of change in GHQ score in intervention and control group 1 and control group 2, adjusted for second job, 1998-1999

| Covariates | ΔGHQ score between 1998-1999 | | | |
| --- | --- | --- | --- | --- |
|  | Control group 1  (Above) | | Control group 2  (Below) | |
|  | (1) | (2) | (3) | (4) |
| Intervention effect on those receiving a wage increase, NMW (yes = 1) | 1.01^*^ | 1.18^*^ | 1.15^*^ | 1.28^*^ |
|  | (0.48) | (0.50) | (0.52) | (0.52) |
| Difference between the control group baseline (the regression constant) and the intervention group in 1998 | -0.40 | -0.54 | -0.37 | -0.49 |
|  | (0.48) | (0.49) | (0.50) | (0.50) |
| Control group baseline in 1998 | 11.4^**^ | 11.5^**^ | 11.4^**^ | 11.4^**^ |
|  | (0.25) | (0.27) | (0.29) | (0.28) |
| Time trend for control group, 1998-1999 | -0.25 | -0.38 | -0.38 | -0.48 |
|  | (0.29) | (0.31) | (0.36) | (0.35) |
|  |  |  |  |  |
| Includes people with second job | Y | N | Y | N |
|  |  |  |  |  |
| Observations | 331 | 312 | 334 | 328 |
| *R*^2^ | 0.0091 | 0.011 | 0.0099 | 0.012 |

*Notes:* Constant included in the model (reported as control group baseline in 1998). Standard errors clustered by individual to correct for repeated observations (intra-individual differences). Higher GHQ score captures better health (1 = worst, 13 = best).

^1^ Service class is the baseline.

* p <0.05, ** p < 0.01

Web Appendix 5: Change in job satisfaction between intervention group and control group 1 (ineligible non-recipients) and control group 2 (eligible non-recipients), 1998-1999.

|  | Difference in means  Intervention-Control group 1 | Difference in means  Intervention-Control group 2 |
| --- | --- | --- |
| Change in job satisfaction between 1998-1999 | -0.37 | 0.066 |
|  | (0.25) | (0.27) |
| p-value | 0.37 | 0.81 |
| n | 170 | 177 |

*Notes:* Standard errors in parentheses. P-value is calculated using two-tailed t-test assuming unequal variances. The intervention group’s new wage was below £4.00. The control remain between £3.60-£4.00

* p <0.05, ** p < 0.01

Web Appendix 6: The probability of becoming unemployed between intervention group and control group 1 (ineligible non-recipients) and control group 2 (eligible non-recipients), 1998-1999.

|  | Difference in means  Intervention-Control group 1 | Difference in means  Intervention-Control group 2 |
| --- | --- | --- |
| Probability of becoming unemployed | -0.017 | 0.0062 |
|  | (0.022) | (0.018) |
| p-value | 0.45 | 0.81 |
| n | 186 | 170 |

*Notes:* Standard errors in parentheses. P-value is calculated using two-tailed t-test assuming unequal variances. The intervention group’s new wage was below £4.00. The control remain between £3.60-£4.00

* p <0.05, ** p < 0.01

Web Appendix 7: Difference-in-difference of change in GHQ score in intervention and control groups, adjusted for overtime premium at 25%, 50%, and 75%, 1998-1999

| **A. Control group 1** | ΔGHQ score between 1998-1999 | | |
| --- | --- | --- | --- |
|  | Overtime premium = 25% | Overtime premium = 50% | Overtime premium = 75% |
|  | (1) | (2) | (3) |
| Intervention effect on those receiving a wage increase, NMW (yes = 1) | 1.03*  (0.43) | 1.02*  (0.45) | 0.97*  (0.46) |
|  |  |  |  |
| Number of observations | 178 | 186 | 177 |
| *R*^2^ | 0.035 | 0.030 | 0.028 |
|  |  |  |  |
| **B. Control group 2** | ΔGHQ score between 1998-1999 | | |
|  | Overtime premium = 25% | Overtime premium = 50% | Overtime premium = 75% |
|  | (1) | (2) |  |
| Intervention effect on those receiving a wage increase, NMW (yes = 1) | 0.99*  (0.47) | 0.92*  (0.46) | 1.03*  (0.51) |
|  |  |  |  |
| Number of observations | 218 | 234 | 197 |
| *R*^2^ | 0.018 | 0.016 | 0.019 |

Notes: Standard errors reported in parentheses. Higher GHQ score captures better health (1 = worst, 13 = best).

* p <0.05, ** p < 0.01

Web Appendix 8: Association between the wage-gap (difference between minimum wage and actual wages) for the intervention group and control group

| 1. **Control group 1** | ΔGHQ score between 1998-1999 |
| --- | --- |
| Wage gap (minimum wage – actual wage) in 1998 (0 = above minimum wage in 1998) | 0.63  (0.53) |
| Number of individuals | 170 |
|  |  |
| 1. **Control group 2** | ΔGHQ score between 1998-1999 |
| Wage gap (minimum wage – actual wage) in 1998 (0 = below minimum wage in 1998-1999) | 0.73  (0.55) |
| Number of individuals | 172 |

*Notes:* Constant estimated in the model (not shown). Control group 1: Comparison of the eligible recipients and ineligible non-recipients. Control group 2: Comparison of the eligible recipients and eligible non-recipients.

Higher GHQ score captures better health (1 = worst, 13 = best). Wage-gap is the minimum wage level in 1999 minus actual wages in 1998.

* p <0.05, ** p < 0.01

Web Appendix 9: Association between receiving the minimum wage and health in control group 1 (A) and control group 2 (B), 1998-1999, with and without restriction on post-intervention wages

| **A. Control group 1** | ΔGHQ score between 1998-1999 | |
| --- | --- | --- |
|  | Post-intervention wages<£4.00 per hour | No restriction on post-intervention wages |
|  | (1) | (2) |
| Intervention effect on those receiving a wage increase, NMW (yes = 1) | 0.93^*^ (0.47) | 0.43 (0.37) |
| Number of individuals | 170 | 313 |
|  |  |  |
| **B. Control group 2** | ΔGHQ score between 1998-1999 | |
|  | Post-intervention wages<£4.00 per hour | No restriction on post-intervention wages |
|  | (1) | (2) |
| Intervention effect on those receiving a wage increase, NMW (yes = 1) | 1.06^*^ (0.52) | 0.80 (0.44) |
| Number of individuals | 172 | 296 |

*Notes:* Constant estimated in the model (not shown). Control group 1: Comparison of the eligible recipients and ineligible non-recipients. Control group 2: Comparison of the eligible recipients and eligible non-recipients.

Higher GHQ score captures better health (1 = worst, 13 = best).

* p <0.05, ** p < 0.01
